# Association of Heat Index and Patient Presentation Rate at a Stadium

**DOI:** 10.5811/westjem.21222

**Published:** 2025-05-19

**Authors:** Jennifer Wolin, Daniel Wolf, John Su, Eric Quinn, David Eng, Humaira Ali, David Lobel, Matt Friedman

**Affiliations:** * Maimonides Medical Center, Department of Emergency Medicine, Brooklyn, New York; † Stanford Medicine, Department of Emergency Medicine, Stanford, California

## Abstract

**Introduction:**

A mass gathering is defined as an event that has the potential to strain the resources of the local health system. An onsite physician for mass gatherings can mitigate the strain on the local health infrastructure. One factor affecting onsite medical usage and patient presentation rates is the heat index, which is a calculated value of perceived heat exposure that combines air temperature and relative humidity. In this study we asked whether there was a positive association between heat index and patient presentation rates for onsite medical care at a bounded (large event in an enclosed location) professional stadium sporting event. We hypothesized that a positive correlation exists between these two variables and assess whether it might surpass current onsite resources.

**Methods:**

We performed a prospective observational study with patients seeking medical care at a baseball stadium in a large northeastern city in the United States. The onsite physician collected information on patients who presented during games held at the stadium. Data on game attendance, temperature in degrees Fahrenheit (F), humidity, and heat index were collected using government and professional organization websites. We assessed the correlation between heat index and patient presentation rate with the Pearson product-moment correlation (PPMC) per 100,000 attendees at the game.

**Results:**

A total of 81 baseball games occurred at the studied stadium from April–September 2023, with eight games excluded due to incomplete data. The heat index ranged from 46°F to 91°F, with a mean (± SD) of 70.8°F (± 10.4°F). The number of patients varied from 0–5 per game, with a mean of 1.92 (± 1.13), and stadium attendance ranged from 25,007 to 47,295, with a mean of 40,824. The patient presentation rate per 100,000 in attendance was 5.04 (± 2.13). The PPMC was calculated to be 0.37 (*P* < .01), indicating a positive correlation between heat index and patient presentation rates. The most common reasons for seeking medical care were lightheadedness and musculoskeletal complaints.

**Conclusion:**

In this study we found that the heat index was moderately associated with higher patient presentation rates at bounded mass gathering baseball events. No additional resources were needed, but this correlation could aid future event medical planning efforts as the climate continues to evolve.

## INTRODUCTION

A mass gathering is defined as an event that has the potential to strain the resources of the local health system or community.^1^–^4^ Understanding patient presentation rates and etiologies at mass gatherings is essential for maximizing the efficient use of resources and onsite health services, and for activating ambulance services. Effective preparedness for mass gatherings can minimize delays to definitive care and alleviate the burden on local hospitals’ resources that would otherwise result from the mass gathering by staffing and planning for predictable health risks, such as heat-related illness. 5–8 Furthermore, data collected from onsite medical care can inform public health surveillance efforts, enabling local emergency departments and 9-1-1 systems to anticipate resource needs and coordinate mutual aid staffing, especially in similarly sized events that may not have onsite medical staffing. Some states have legislated certain regulatory criteria for providing healthcare services at such events to ensure sufficient mass-gathering medical care. In New York State, for example, any event with greater than 5,000 attendees requires a physician to be available within 15 minutes, while events with greater than 30,000 attendees require a physician onsite.^9^

Event physicians can enhance the medical care given by prehospital personnel by providing on-scene medical direction and offering independent and complex medical decision-making, including the ability to return participants safely to the event without unnecessary transport.^10^ For those who may require medical evaluation, the onsite physician can determine the most optimal transport mode or even consent for an “against medical advice” decision, if appropriate.^11^ This allows for optimizing transport resources and minimizing the impact on local ambulance services and emergency departments.^10^ Event physicians may also have prehospital training, encompassing mass casualty triage and familiarity with the incident command system, to effectively manage a sudden surge of patients.^11^ The exact role also varies based on the event and distance to definitive care. Events located hours from the closest hospital require more extensive onsite medical care to return participants to the event and conserve medical transport resources due to long turn-around times.

Most mass gathering literature defines three domains impacting healthcare at events.^12^ These include biomedical (spectator demographics and health status), psychosocial (crowd behavior, culture, reason for attendance), and environmental (characteristics of venue, weather, presence of drugs or alcohol). The biomedical domain includes the principles of patient presentation rates (PPR) and transfer to hospital rates (TTHR).^13^–^14^ The PPR provides insight into the utilization of onsite health services and refers to the number of attendees who present requesting onsite medical care, while TTHR provides information on prehospital ambulance transports out of the venue. On occasion, patients may refuse ambulance services and elect to self-transfer. This is known as the self-transfer to hospital rate (STHR), which is defined as the number of patients who present for medical attention and elect to go to a hospital by private means. The total number of patients who present to local hospitals for medical care by any means is known as the referred to hospital rate (RTHR).^14^

**Population Health Research Capsule**

What do we already know about this issue?

*Heat increases medical needs at mass gatherings, but data on heat index and its impact on patient presentation rates (PPR) is limited.*

What was the research question?

*Is there a positive association between heat index and PPR at a bounded professional baseball stadium event?*

What was the major finding of the study?

*For every 10°F rise in heat index, PPR rates increased by 1.46 per 100,000 attendees (r = 0.37, P < .01).*

How does this improve population health?

*These findings help optimize medical staffing at bounded mass gatherings, ensuring sufficient resources as climate change increases heat-related health risks.*

Prior research on PPR has produced a wide range of values depending on the nature of the event. For example, an annual, large outdoor music festival in Austria recorded a median PPR of 12.01 per 1,000 attendees.^15^ In contrast, the 2019 Rugby World Cup in Japan reported a considerably lower rate with a PPR of 2.63 per 10,000.^16^ The New York State Fair demonstrated an annual PPR of 4.8 per 10,000 attendees.^17^ Another study exploring various event types found PPRs per 10,000 to be 4.85 at baseball games, 6.75 at football games, and 30 per 10,000 at rock concerts.^18^ At the South Africa FIFA World Cup, the PPR was 6.6 per 100,000.^19^

Heat is one important environmental factor affecting PPR and TTHR, leading to various heat-related illnesses; it is on the rise worldwide.^20^–^21^ This is most accurately captured by the heat index, which is a calculated value of perceived heat exposure, combining air temperature and relative humidity. The heat index can help better stratify the risk of heat-related illnesses.^22^ This ranges from mild heat cramps to more severe, life-threatening conditions such as heat exhaustion and heat stroke.^23^ Several studies have explored the relationship between PPR and temperature; however, few studies look at PPR in the context of the heat index, which provides a more accurate representation of heat’s effect on the human body.^24^–^25^ Perron et al demonstrated that PPR was tied to the heat index at college football games in the southeastern United States during the fall and winter months.^25^ This study examined whether the correlation persists during summer months by analyzing the impact of heat on patient presentation rates and its potential to strain onsite medical resources and local healthcare systems. We hypothesized that a positive correlation exists between PPR and heat index at large baseball events in a bounded stadium in the northeastern US but that this increase in patients would remain within the capacity of onsite resources.

## METHODS

This prospective, observational study examined the cases of attendees seeking medical attention during baseball games in a large northeastern city in the US. These games took place outdoors in a bounded stadium with ticketed seats. The stadium has open-air seating and limited natural shade with much of the seating areas exposed. The region’s climate is characterized as humid subtropical with summer temperatures reaching as high as 100° Fahrenheit (F).^26^ The stadium in this study can accommodate 47,309 spectators.

We collected data during regular season games at the stadium between April–September 2023. Patrons could self-present or be brought by emergency medical technicians (EMT) via mobility assist devices and were met by the paramedic supervisor at the front of the medical office. We excluded patrons who self-presented to the medical office solely asking for over-the-counter items such as bandages, acetaminophen, or non-steroidal anti-inflammatory drugs. We included only individuals with acute medical complaints who were then directed for evaluation by a physician. To prevent bias and capture cases of occult heat illness, exacerbations of chronic illness due to heat, and potential sequelae from heat-related crowd behavior, we included all medical complaints.

At these events, the responsibility of the onsite physician was to assess the patient, provide initial stabilization, and determine the need for ambulance transport to a nearby hospital. The onsite physician was an emergency physician with medical command training from the surrounding area. The onsite physician gathered data during home games, using Research Electronic Data Capture (REDCap) tools hosted at the Maimonides Medical Center to record patient information such as age, sex, chief complaint, treatments administered, physician diagnosis, and disposition. Non-physician staffing included one EMS supervisor, two paramedics, five EMTs, and one dedicated transport unit. The EMTs were dispersed throughout the crowd to help facilitate patients in reaching the medical office and then returned to continue crowd surveillance after patient handoff.

We obtained the total number of attendees at each game from Baseball-Reference.com. Temperature and humidity data was obtained from Time and Date AS (Stavanger, Norway), with the game start time as the reference point. We then used this data to calculate the heat index using the US National Oceanic and Administration Weather Prediction Center (College Park, MD). Results are reported with descriptive and quantitative statistics. The correlation between heat index and patient presentation rate was assessed with the Pearson product-moment correlation (PPMC). The PPMC is a statistical tool that we used to control stadium attendance as a contributing factor to the overall number of patients seen; we assessed for a positive correlation between the heat index and the ratio of the number of patients cared for per 100,000 attendees at the game. In addition, we performed chi-squared testing to assess for any statistical difference between sexes and final disposition among the chief complaints and treatments rendered.

## RESULTS

**Figure 1:**
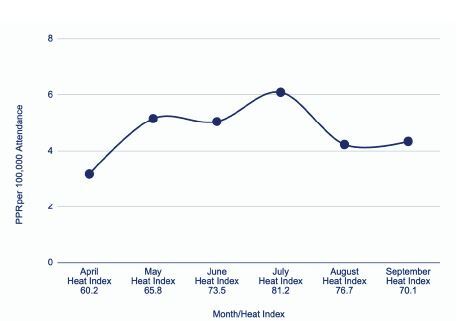
Mean patient presentation rate by month. *PPR*, patient presentation rate.

A total of 81 home baseball games occurred from April–September 2023, with eight games excluded due to incomplete data from lack of documentation. There were 92 patient encounters among the 2,926,363 total attendees. The heat index ranged from 46°F–91°F, with a mean (±SD) of 70.8°F (± 10.42°F). The number of patients per game varied from 0–5, with a mean of 1.92 (±1.13), and stadium attendance ranged from 25,007 to 47,295. The PPR in our study is defined as the number of patient presentations per 100,000 attendees. The PPR across all games was 5.04 ± 2.13, with the highest PPR in July (6.09 ± 1.66) and the lowest in April (3.19 ± 1.13) (Figure 1).

**Figure 2:**
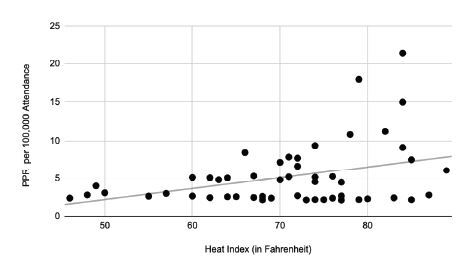
Correlation between heat index and patient presentation rate by degrees in Fahrenheit. *PPR*, patient presentation rate.

The average patient age for those seeking medical care was 39.9 years. We calculated the PPMC to be 0.37 (*P* < .01), indicating a moderately positive correlation between heat index and PPR. Our linear equation demonstrated that for every 10-degree increase in heat index, there was a 1.46 increase in the number of patients requiring emergency care per 100,000 attendees (Figure 2).

The mean TTHR was .37 per 100,000 attendees with a peak in June of .60 and a trough in August of .12. The STHR per 100,000 attendees across all games was 0.3 ± 0.6, and our mean RTHR across all games was .67 ± 0.7 (Table 1).

Chief complaints were broken down by category. Among patients seeking medical attention, the most frequent complaint was musculoskeletal, affecting 31 patients (34%). This was followed by lightheadedness in 24 patients (26%) and nine patients (10%) who were intoxicated. There were no cardiac arrests in this cohort. These chief complaints were comparable in frequency between male and female patients, as well as between patients transported to the hospital vs those who returned to the event (Table 2). A total of 108 treatments were documented across all games because some patients received multiple treatments. The most common treatments included 33 ice packs (31%), 21 (19%) administered NSAIDs or acetaminophen, 12 (11%) miscellaneous, and 11 patients (10%) who received oral fluids. Two patients received an electrocardiogram (2%), and five received intravenous fluids (5%).

Treatments rendered similarly had comparable distributions between sex and final dispositions, and no statistical difference was observed (Table 3). Of all patients who presented, 32 (35%) were referred to the hospital., an average of fewer than one patient per game. Fourteen patients elected to refuse transport by EMS and chose to transport themselves by private vehicle, and 18 (19.2%) who presented for physician evaluation were consequently transported via onsite ambulance. There were zero instances where ambulances from the local 9-1-1 system were needed to transport patients from the event.

## DISCUSSION

In this study we examined the correlation between PPR and the heat index at a mass-gathering baseball event. We found a moderate positive correlation between the heat index and PPR. The PPMC was 0.37 (*P* < .01) with a linear equation demonstrating that there was a 1.46 increase in the number of patients presenting for emergency care per 100,000 attendees for every 10-degree increase in the heat index. This could help inform future planning efforts, especially as the host city is projected to have a tripling of days above 90°F by the year 2050.^27^ During the study period, there were no instances of outside resources used to render medical aid at the event.

Comparing our event to other mass gatherings is challenging because so many studies depict specific events with unique characteristics. It would be potentially inaccurate to make comparisons to non-sporting events due to different crowd behavior and milieu characteristics. In a study involving spectators at a bounded football game, Perron et al found that for every 10-degree increase in heat index, three more patrons per 10,000 attendees would present for care.^25^ Our findings were most similar to the 2010 South Africa World Cup, which shared key characteristics with our event. Both stadium events had ticketed entry, offered alcohol for sale and free water, and resulted in similar medical needs and PPR (5.04 vs 6.6 per 100,000). However, other well-studied events, such as college football games held in similar settings but with different demographics and environmental traits, revealed significantly higher PPRs.^28^ This suggests that factors beyond venue characteristics, such as crowd mood and behavior, may influence the PPR at mass gatherings.

Of all patients who presented for medical care, an average of .67 per 100,000 attendees were referred to nearby hospitals, which likely did not impact operations at local hospitals. Of the 92 patients who presented, 19.2% were transported via ambulance. In months with relatively low heat indexes such as April and May, a single patient was transported via ambulance in one of every three games. However, in months with higher heat index such as June and July, this increased to an ambulance transport every two games. This increase, however, did not meaningfully stress the ability to provide medical aid using our current onsite staffing model.

Overall, the data suggesst a relatively low and predictable PPR at baseball events with a low RTHR and TTHR. However, when compared to similar studies, there is a much higher TTHR in our setting than in others. Patients with the chief complaint of “lightheadedness” were disproportionately more likely to be transported to the hospital, making up 28% of chief complaints but 38% of all transports. In our study, 19.2% of patients were taken to a hospital by ambulance vs 5.7% in Perron et al and 4.1% in Hardcastle et al.^19^,^25^ Perron et al found a significantly higher PPR, while only 5.7% of patrons required ambulance transports. Our study also had a higher average patient age than the other referenced studies. Compared to Hardcastle et al, we recorded a mean patient age that was eight years older. Although patient acuity may explain the difference in TTHR, these events also occurred in different countries. The economic burden of paying for ambulance services, and differences in professional liability, may also account for the difference in TTHR.

We found the most common medical complaints were musculoskeletal followed by lightheadedness. There were no differences in chief complaints between sexess or in the final disposition of the patient. This aligns with other studies on mass gatherings where the predominant complaints were musculoskeletal. In the study by Hardcastle et al, of their 316 presentations, 98 (31%) were also musculoskeletal or minor trauma.

There were 108 documented interventions; some patients received multiple interventions while others received none. The physician treated patients with various interventions, with the most common being ice packs (31%), corresponding to 34% of complaints being musculoskeletal. Pain relievers such as NSAIDs and acetaminophen were also frequently used. Oral fluids were the third most common intervention; this is likely under-reported as many patients brought their own water containers.

Interestingly, a limited number of ECGs and IV fluid boluses were administered even though Advanced Life Support was available at all events. This suggests that most patients who presented for medical evaluation did not require advanced prehospital intervention and were likely low-acuity patients. There were zero cases of cardiac arrest during the study period. Removing patients from the heat and providing rest helped most recover. The final disposition of patients was similar for each treatment group.

## LIMITATIONS

Our study has several limitations. Because it was an observational study with a 10% missingness rate, we could only establish a correlation between heat index and patient presentation rates and not causation. The data abstractors were not blinded to the premise of the study. Additionally, only one baseball season was studied, which limited the climate and patient data to one specific year. Because our analysis was limited to a single season, we could not further isolate games with higher heat indices without significantly reducing the sample size. Due to the modest sample size, there is a potential for variability, raising concerns about the robustness of the findings. While a moderate correlation was observed for PPR, no such relationship was found for TTHR. For instance, despite having a similar heat index to June, August had a much lower TTHR and PPR.

We chose not to include patients seeking OTC medication who didn’t request to see a physician in order to isolate physician staffing needs and the effect of heat index on patient presentation for medical usage. However, the definition of what constituted a patient encounter likely varied between physicians and resulted in a more heterogeneous population despite our efforts to define it. Some treatment interventions may have been undercounted, such as giving oral fluids to patients. We did not capture data on the total supply usage, such as OTC medications dispensed without physician evaluation. Capturing total supply usage may also be of interest to event directors. Lastly, our high TTHR (19.2%) may reflect how we defined a patient. Unlike other studies that classified all individuals who presented as patients, regardless of physician evaluation, we only included those who were formally evaluated by a physician. This measurement difference contributed to our higher TTHR.

While our study shows more patients needing care with a higher heat index, this cannot be extrapolated below 46° F, which was the study’s lowest recorded temperature. Studies suggest that extremely cold temperatures also increase PPR given its association with cold-related medical conditions.^28^–^29^ In addition, wedid not us Wet Bulb Globe Temperature (WBGT), which is another modality for temperature measurement that takes into account factors such as humidity, wind, and sunlight.^30^–^33^ While not used before for predicting medical needs at spectator sporting events, WBGT is used in active participation event studies, and future research could explore this further, providing a more comprehensive understanding of variables within the environmental domain.

## CONCLUSION

We found that the heat index was moderately associated with higher patient presentation rates at bounded mass-gathering baseball events. Although no additional onsite resources were needed, likely due to low baseline presentation rates and moderate weather during the study period, this correlation could help inform event medical planning efforts as the climate continues to evolve.

## Figures and Tables

**Table 1:** Medical usage rates by month.

Month	Mean number of patients per game	Mean STHR	Mean TTHR via ambulance	Mean RTHR	Stadium attendance	Mean stadium capacity filled	Mean heat index in degrees Fahrenheit	Mean total patients per 100,000 attendees (PPR)	Mean EMS transports per 100,000 attendees
April	1.27 ± 0.46	0.08 ± 0.44	0.36 ± 0.50	0.44 ± 0.50	39,765 ± 7,106	0.85 ± 0.15	60.2 ± 8.44	3.19 ± 1.13	0.88 ± 1.25
May	2.00 ± 0.69	0.10 ± 0.67	0.21 ± 0.69	0.31 ± 0.82	38,694 ± 2,132	0.83 ± 0.04	65.8 ± 10.31	5.16 ± 1.95	0.55 ± 0.94
June	2.20 ± 1.68	0.11 ± 0.53	0.60 ±0.69	0.71 ± 0.59	43,511 ± 3,311	0.93 ± 0.07	73.5 ± 4.50	5.05 ± 1.27	1.32 ± 1.52
July	2.66 ± 1.50	0.66 ± 0.81	0.50 ± 0.54	1.16 ± 0.98	43,612 ± 3,433	0.93 ± 0.07	81.2 ± 4.73	6.09 ± 1.66	1.17 ± 1.29
August	1.75 ± 1.03	0.75 ± 0.70	0.12 ± 0.35	0.87 ± 0.64	41,316 ± 3,130	0.88 ± 0.06	76.7 ± 4.68	4.23 ± 2.45	0.29 ± 0.83
September	1.67 ± 0.81	0.50 ± 0.83	0.16 ± 0.40	0.66 ± 0.81	38,472 ± 3,209	0.82 ± 0.06	70.1 ± 9.62	4.34 ± 2.10	0.44 ± 1.08
Mean ± SD	1.92 ± 1.13	0.30 ± 0.61	0.37 ± 0.56	0.67 ± 0.73	40,824 ± 4,550	0.87 ± 0.09	70.8 ± 10.42	5.04 ± 2.13	0.90 ± 1.39

Medical usage rates by month include mean number of patient presentations per game, transport rate to hospital via ambulance, and referred-to-hospital rate.

*STHR*, self-transport to hospital rate; *TTHR*, transport to hospital rate; *RTHR*, referred to hospital rate; *PPR*, patient presentation rate; *EMS*, emergency medical services.

**Table 2:** Overview of chief complaints.

	Total (N = 92)		Male (n = 54)		Female (n = 38)		Significance	Referred to hospital (n = 39)		Back to event (n = 53)		Significance

Chief complaint	n	%	n	%	n	%	p	n	%	n	%	p
Abdominal pain	6	7	3	6	3	8	0.97	4	10	2	4	0.97
Allergic reaction	6	7	3	6	3	8	0.97	2	5	4	8	0.94
Chest pain	5	5	2	4	3	8	0.93	2	5	3	6	0.93
Intoxication	9	10	6	11	3	8	1	2	5	7	13	0.96
Miscellaneous	7	8	2	4	5	13	0.95	0	0	7	13	0.5
Musculoskeletal	31	34	19	35	12	32	1	13	33	18	34	1
Shortness of breath	4	4	0	0	4	11	0.5	1	3	3	6	0.8
Lightheadedness	24	26	19	35	5	13	1	15	38	9	17	1

**Table 3:** Overview of treatments rendered to patients who presented for medical care.

	Total (N=108)		Male (n=56)		Female (n = 52)		Significance	Referred to hospital (n=45)		Back to event (n=63)		Significance

Treatment rendered	n	%	n	%	n	%	p	n	%	n	%	p
Bandage	8	7	5	9	3	6	0.99	3	7	5	8	0.98
Diphenhydramine	6	6	3	5	3	6	0.97	3	7	3	5	0.97
Albuterol nebulizer	4	4	3	5	1	2	0.9	1	2	3	5	0.8
ECG	2	2	1	2	1	2	0.75	1	2	1	2	0.75
Ice pack	33	31	17	30	16	31	1	14	31	19	30	1
Intravenous fluids	5	5	3	5	2	4	0.95	3	7	2	3	0.95
NSAIDs/Acetaminophen	21	19	11	19	10	19	1	9	20	12	19	1
Oral Fluids	11	10	5	9	6	12	1	4	9	7	11	1
Ondansetron	6	6	4	7	2	4	0.97	2	4	4	6	0.94
Miscellaneous	12	11	4	7	8	15	1	5	11	7	11	1

*ECG*, electrocardiogram; *NSAID*, nonsteroidal anti-inflammatory drug.
